# Synergistic Antibacterial Activity of Benzalkonium Bromide and Cu-Bearing Duplex Stainless Steel against *Pseudomonas aeruginosa*

**DOI:** 10.3390/microorganisms11030711

**Published:** 2023-03-09

**Authors:** Xiaomeng Liu, Chengshuo Qiu, Mingxing Zhang, Enze Zhou, Dake Xu, Yongqiang Fan, Yongbo Song

**Affiliations:** 1 College of Life and Health Sciences, Northeastern University, Shenyang 110819, China; 2 Shenyang National Laboratory for Materials Science, Northeastern University, Shenyang 110819, China; 3 Electrobiomaterials Institute, Key Laboratory for Anisotropy and Texture of Materials (Ministry of Education), Northeastern University, Shenyang 110819, China; 4 Department of Life Sciences and Biopharmaceuticals, Shenyang Pharmaceutical University, Shenyang 110016, China

**Keywords:** *Pseudomonas aeruginosa*, benzalkonium bromide, antibacterial, 2205-Cu DSS

## Abstract

The bactericide benzalkonium bromide is widely used to kill *Pseudomonas aeruginosa,* which causes microbiologically influenced corrosion (MIC). However, the extensive use of benzalkonium bromide will enhance bacterial drug resistance and cause environmental pollution. In this study, benzalkonium bromide combined with Cu-bearing 2205 duplex stainless steel (2205-Cu DSS) was used to kill *Pseudomonas aeruginosa*; the germicidal rate of the combination of benzalkonium bromide and 2205-Cu DSS was 24.2% higher than that of using benzalkonium bromide alone, after five days. The antibacterial efficacy was evaluated using an antibacterial test and biofilm observation. The results showed that, in the presence of *P. aeruginosa*, the combination of 23.44 ppm benzalkonium bromide and 2205-Cu DSS showed the best antibacterial efficacy.

## 1. Introduction

*Pseudomonas aeruginosa* is a Gram-negative bacterium that is widely distributed in nature [1,2,3], and it causes metal corrosion by forming a biofilm on the material’s surface [4,5]. It is one of the essential microorganisms associated with corrosion in marine and soil environments [6]. *P. aeruginosa* is usually resistant to bactericides once it forms a biofilm [7]. The long-term and widespread use of bactericides leads to the increased resistance of *P. aeruginosa*.

Benzalkonium bromide is a non-oxidizing bactericide that has been popularly used in sterilization, oilfield water injection, algae control, antisepsis, and so on [8,9]. Benzalkonium bromide can change the permeability of cell membranes, make intracytoplasmic fluid exosmosis, hinder metabolism, and achieve the bactericidal effect [10,11].

Duplex stainless steel is popularly used in the marine industry, in water circulation systems, and in the petroleum industry as a result of its good mechanical strength and corrosion resistance [12,13]. The corrosion resistance of stainless steel mainly depends on the passivation film formed on the surface [14]. The passive film can protect metals against uniform corrosion but is still susceptible to microbiologically influenced corrosion (MIC) [15]. It is reported that the global cost of corrosion exceeds USD four trillion, of which microbial corrosion accounts for more than 20% [16]. A disinfectant that has been used for a long time is copper. Its bacterial inhibitory effect depends on the release of Cu^+^ and Cu^2+^ near the copper surface [17,18,19]. 2205-Cu stainless steel is a new antimicrobial metal material that forms the copper-rich phases during the solution and aging treatments.

It has been reported that 2205-Cu duplex stainless steel (2205-Cu DSS) is resistant to the MIC of *P. aeruginosa* in the marine environment [20]. This study found that the combination of benzalkonium bromide with 2205-Cu DSS greatly enhanced antibacterial activity, in which the concentration of benzalkonium bromide was only 23.44 ppm. This work provides a novel strategy to improve the antimicrobial activity of metal materials and achieves a better bacterial killing efficiency by using a lower concentration of benzalkonium bromide, which is expected to contribute to alleviating environmental pollution and to decreasing the risk of super-bug development.

## 2. Materials and Methods

### 2.1. Materials

The traditional 2205 duplex stainless steel (2205 DSS) used in this study was purchased from Taiyuan Iron and Steel Group. The Cu-bearing 2205 DSS was obtained from Yang Ke Research Group, Institute of Metals, Chinese Academy of Sciences. Table 1 shows the chemical compositions (wt%) of the 2205 DSS and 2205-Cu DSS specimens, which were provided by the manufacturer. The specimens were annealed at 1200 °C for 1 h and then aged at 540 °C for 4 h. The 2205 DSS and 2205-Cu DSS coupons (a small piece of metal), with an exposed surface area of 1.0 cm × 1.0 cm, were rubbed with different grades of silicon carbide papers (200, 400, 600, 800, 1000 grits), cleaned with absolute ethanol, air-dried, and sterilized under UV lights for 20 min before being immersed in the culture medium.

### 2.2. Bacteria and Bactericide

*P. aeruginosa* MCCC 1A00099 was purchased from the Marine Culture Collection of China (MCCC), Xiamen, China. The LB culture medium (Tryptone 10 g/L, Yeast extract 5 g/L, NaCl 10 g/L, pH 7.2) was used to culture the bacteria in this study, The culture medium was autoclaved at 121 °C for 20 min. Benzalkonium bromide was purchased from Sigma-Aldrich with a purity of ≥99.0%. A concentration of 200 mg/mL stock was prepared, and the minimum inhibitory concentration of benzalkonium bromide against *P. aeruginosa* was tested.

### 2.3. Colony-Forming Unit (CFU) Counting

The 2205 DSS or 2205-Cu DSS coupons were immersed in the bacterial culture (10^6^ cfu/mL) for 1, 3, and 5 days, respectively. The samples were taken out and washed with phosphate buffer (PBS) three times until the planktonic bacteria were removed clearly. The bacteria on the coupon surface were harvested by whirlpool oscillation, serially diluted, and plated on the LB medium plate. The CFUs were counted after 24 h incubation at 30 °C.

### 2.4. Visualization of Live/Dead Cells in Biofilms

The coupons immersed in bacterial culture (10^6^ cfu/mL) for 1, 3, and 5 days were taken out, washed with PBS three times, placed in 2 mL PBS, and incubated with 20 μL SYTO-9 and PI (propidium iodide) dye for 20 min. The live/dead cell was distinguished by checking the fluorescence with confocal laser scanning microscopy (CLSM) (Model C2 Plus, Nikon, Tokyo, Japan). Live cells emitted green fluorescence under 488 nm excitation light, while red fluorescence for dead cells was observed at 559 nm excitation light.

### 2.5. Surface Morphology of Biofilm

The coupons immersed in bacterial culture (10^6^ cfu/mL) for 1, 3, and 5 days were taken out and washed with PBS three times. They were incubated with 2.5% glutaraldehyde at 4 °C for 4 h. Dehydration was carried out with 50%, 60%, 70%, 80%, 90%, 95%, and 100% ethanol. After the ethanol evaporated and dried, the samples were sprayed with gold and observed under scanning electron microscopy (SEM) (Field emission scanning electron microscope, FESEM. Ultra-Plus, Zeiss, Oberkochen, Germany).

### 2.6. Flow Cytometry (FCM)

The coupons immersed in bacterial culture (10^6^ cfu/mL) for 1, 3, and 5 days were taken out and washed with PBS three times. Then the cells on the coupon surface were collected in a 50 mL LB culture medium. Thirty-five microliters of Mycolight^TM^ 520 dye was added to the medium and incubated with cells at 35 °C for 20 min under light avoided. The impurities were removed from the bacterial sample with a 500-mesh screen, and the cell number was quantitated by flow cytometry (FCM) (CyFlow Cube 8, Partec, Görlitz, Germany).

### 2.7. Real-Time Cell Analyzers (RTCA)

The coupons immersed in bacterial culture (10^6^ cfu/mL) for 1, 3, and 5 days were taken out and washed with 2 mL PBS two times. The coupons were shaken in 1 mL PBS solution to collect the bacteria. One hundred microliters of well-mixed cell suspension was transferred to the E-plate 16. After being incubated at room temperature for 30 min, the E-plate 16 was placed onto the RTCA SP Station located inside the incubator to record the continuous impedance. The CI value changed proportionally when the cell was attached to the electrode [21].

### 2.8. ATP Bioluminescence Assay

The ATP of different groups was quantified by the bioluminescence method to evaluate the total number of sessile bacteria. After 1 day, 3 days and 5 days of treatment, UPF-10-ATP (Youpu, Beijing, China) was used for analysis, expressed in relative light units (RLU) [22].

### 2.9. Statistical Analysis

All of the statistical analysis for the data was performed using GraphPad Prism 8 according to analysis of variance (ANOVA) with the application of Tukey’s multiple comparison test.

## 3. Results

### 3.1. The pH Value Did Not Change Significantly in the Presence of Bactericide

**Figure 1:**
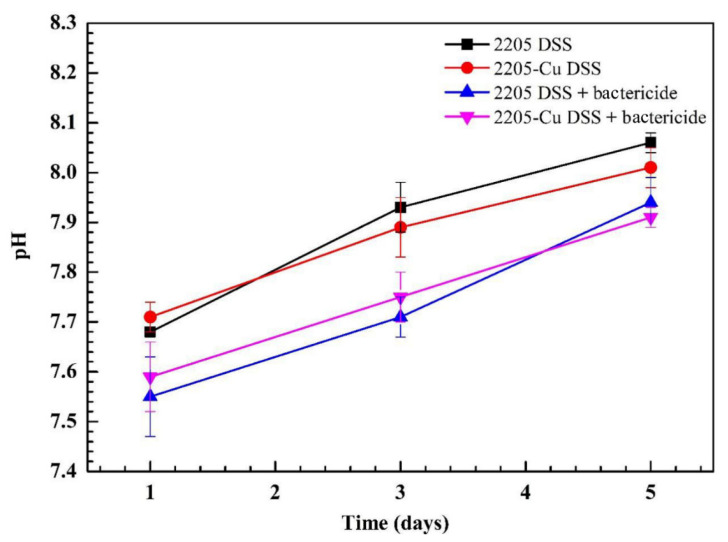
The pH values in different groups in the presence of *P. aeruginosa*. The bactericide is benzalkonium bromide. (*n* = 3).

In order to rule out the effect of the pH change due to the addition of bactericide on the bacteria growth, we measured the pH value. As shown in Figure 1, there was no significant difference in the pH between the control group (2205 DSS) and the test group (2205-Cu DSS) without bactericide, which was approximately 7.7 on day 1, 7.9 on day 3, and 8.0 on day 5. The pH values of the 2205 DSS + bactericide group and the 2205-Cu DSS + bactericide group supplemented with bactericide were similar, at about 7.6 on day 1, 7.7 on day 3, and 7.9 on day 5. The pH values of the samples with bactericide did not change significantly, compared with those without bactericide, and we can rule out that the pH change caused by the bactericide would kill the bacteria.

### 3.2. Antibacterial Effect

**Figure 2:**
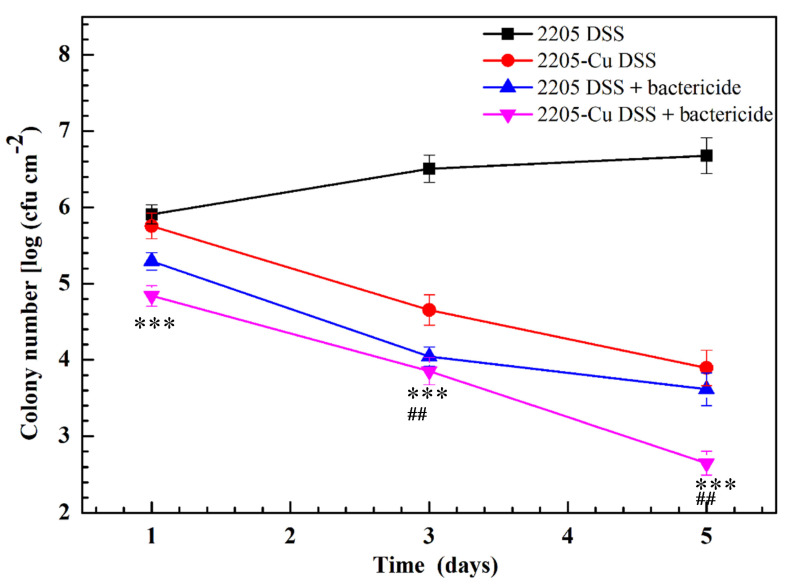
Colony numbers of the biofilms on 2205 DSS and 2205-Cu DSS after the 1-, 3-, and 5-day incubations. The bactericide is benzalkonium bromide. *** *p* < 0. 001 (sample vs. the 2205 DSS group); ## *p* < 0.01 (sample vs. the 2205-Cu DSS group). (*n* = 3).

The CFU counting method was adopted to measure the antimicrobial effects. Figure 2 shows that the number of bacteria adhering to the surface of the control group (2205 DSS) increased continuously within 5 days of incubation. The CFUs of bacteria adhering to the surface after 5 days of culture were (9.3 ± 4.8) × 10^6^ cell/cm^2^. In comparison, the CFUs of bacteria adhering to the surface decreased for the test groups. After 5 days of culture, the CFUs of bacteria adhering to the surface were (2.5 ± 3.7) × 10^4^ cell/cm^2^, (8.1 ± 3.3) × 10^3^ cell/cm^2^, and (6.6 ± 2.4) × 10^2^ cell/cm^2^ for 2205-Cu DSS, 2205-DSS+ bactericide, and 2205-Cu DSS + bactericide, respectively. The CFUs of the test group 2205-Cu DSS + bactericide were the smallest after 5 days, which exhibited the best antimicrobial activity. The results indicated that the combination of 2205-Cu DSS and the bactericide was more effective than the single use of either 2205-Cu DSS or the bactericide. The germicidal efficacy was verified by the ATP bioluminescence assay at the same time (Figure S1).

### 3.3. Morphology of Biofilm

**Figure 3:**
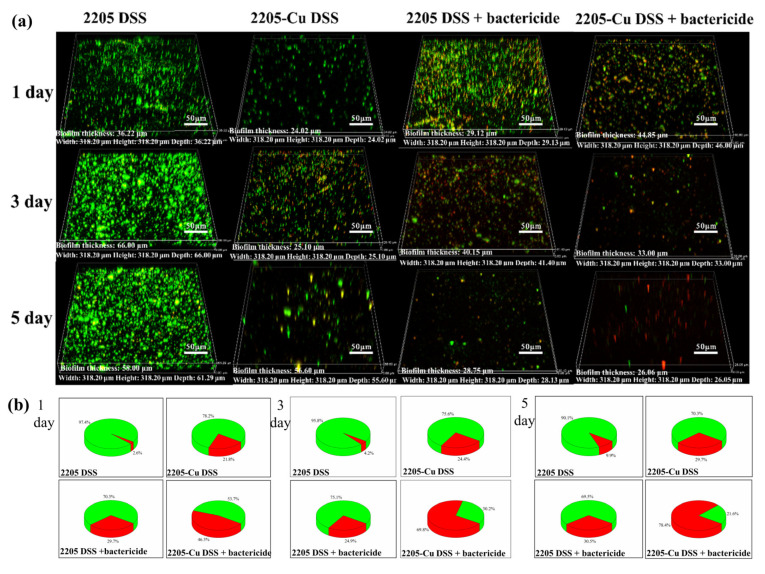
(**a**) CLSM images (3D) of the biofilms on 2205 DSS and 2205-Cu DSS after the 1-, 3-, and 5-day incubations. The bactericide is benzalkonium bromide. (**b**) The proportion of live/dead sessile cells. Red represents dead cells; green represents live cells.

The live and dead cells were stained by SYTO-9 and PI, respectively, which showed a green fluorescence and a red fluorescence. The live/dead cells on the coupon surface after 5 days of incubation were checked by SYTO-9 and PI staining. Figure 3a shows that the number of live bacteria in the control group (2205 DSS) increased, and almost no dead bacteria appeared. In the test group (2205-Cu DSS), a few dead bacteria were observed on the first day, the number of dead bacteria increased on day 3, and the total number of bacteria decreased because of the exfoliation of dead bacteria on day 5. Compared with the 2205 DSS, the 2205-Cu DSS with the bactericide exhibited much fewer live sessile cells (green dots) on the coupon surface. Figure 3b shows the proportion of live and dead sessile cells. The decreased number of live cells indicated that the antibacterial effect of 2205-Cu DSS in combination with benzalkonium bromide was more effective.

**Figure 4:**
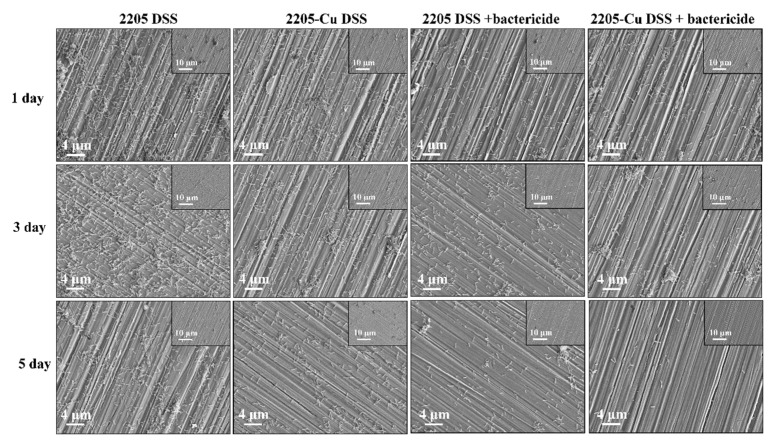
SEM biofilm images on coupons of 2205 DSS and 2205-Cu DSS after the 1-, 3-, and 5-day incubations.

SEM was used to observe the bacteria attachment. Figure 4 shows the bacterial concentration on the surface of the control group (2205 DSS) after 1, 3, and 5 days of incubation. The test group (2205-Cu DSS + bactericide) had the least number of bacterial cells on the surface on day 5.

### 3.4. Survival Rate

**Figure 5:**
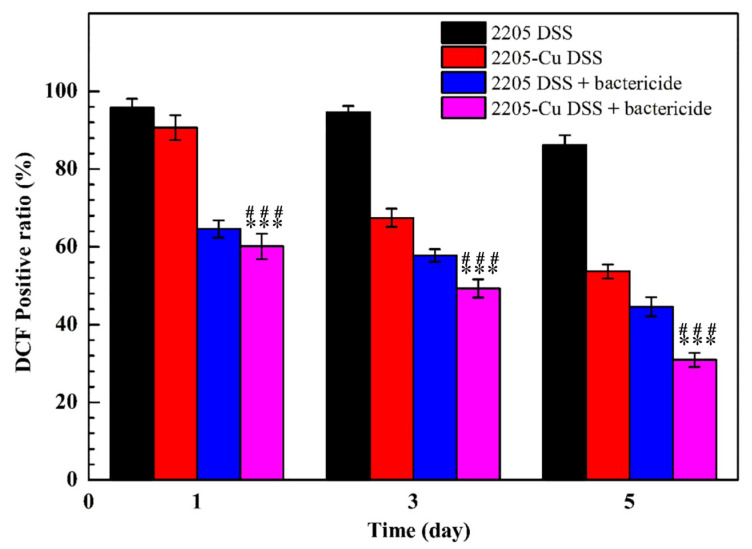
Adherent bacteria survival rate of different groups. *** *p* < 0.001 (sample vs. the 2205 DSS group); ### *p* < 0.001 (sample vs. the 2205-Cu DSS group). (*n* = 3).

The antimicrobial effect reduced both the bacteria and the survival rate of the bacteria attached to the sample surface. The MycolightTM 520 dye was used to label the live bacteria, which was positive for the test results, and the dead bacteria without labeling were negative. As shown in Figure 5, the positive rate of the control group (2205 DSS) remained above 90%, at 90.8%, 69.3%, and 66.1% on days 1, 3, and 5 for the test group (2205-Cu DSS), respectively. The positive rates of the test group 2205 DSS + bactericide and the test group 2205-Cu DSS + bactericide were 65.4% and 60.5% on day 1, respectively, 61.7% and 52.6% on day 3, respectively, and 44.3% and 30.8% on day 5, respectively. The survival rate of the bacteria in the 2205-Cu DSS combined with the bactericide group was the lowest.

**Figure 6:**
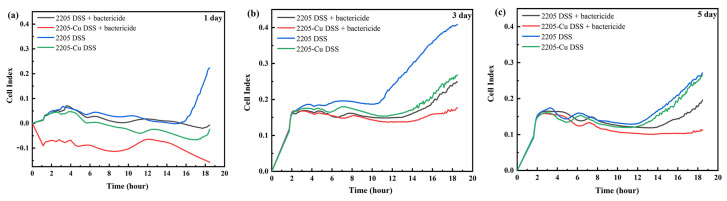
The cell growth curves established by cell impedance measurement under different groups cultured with *P. aeruginosa* cell concentration of 10^6^ cells/mL for 1, 3, and 5 days.

After *P. aeruginosa* cell adhesion and multiplication, the cell index (CI) increased at the stage of cell growth. The impedance curve represented the cell growth curve. After incubation for approximately 12 h, the CI value of the control group increased rapidly. In comparison, the CI value of the test group (2205 Cu DSS + bactericide) remained stable or decreased slightly (Figure 6). The results demonstrated that there was almost no growth of cells attaching to the 2205 Cu DSS surface with the bactericide treatment.

## 4. Discussion

Corrosion caused by *P. aeruginosa* is reported in many fields, such as the industrial and marine environments [6,23]. As such, it is crucial to develop new antimicrobial strategies [24,25]. In this work, the combination of 2205-Cu DSS with benzalkonium bromide exhibited excellent antibacterial performance against *P. aeruginosa*.

**Scheme 1:**
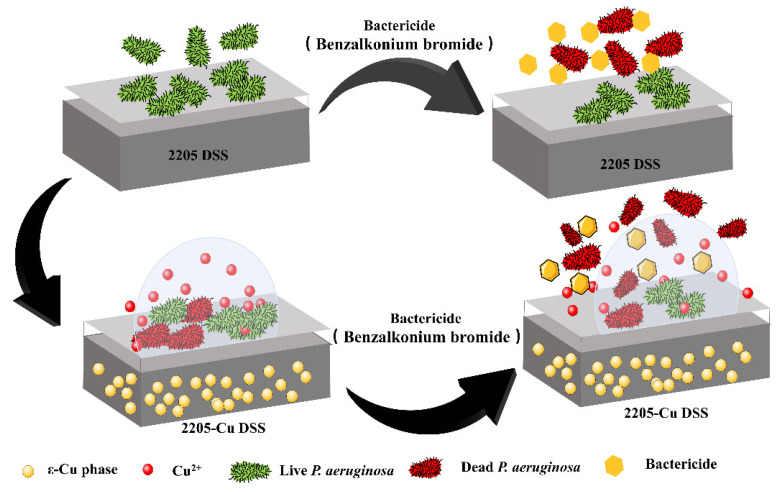
Schematic illustration of the antimicrobial mechanism used with 2205-Cu DSS and bactericide.

Currently, the antibacterial mechanism of copper-containing materials mainly depends on the release of Cu^+^ and Cu^2+^, which are close to the surface. The copper ion has a wide-spectrum germicidal capability. Duplex stainless steel containing copper is a new kind of antibacterial material that will precipitate the copper-rich phase after a solid solution and aging process [17]. Previous studies have shown that copper-containing stainless steel could inhibit the growth of *P. aeruginosa* [26]. Scheme 1 illustrates the antibacterial mechanism of the combination of 2205-Cu DSS with bactericides. Copper ions are continuously released from the 2205-Cu DSS matrix [18], which inhibit or kill the growth of sessile cells, resulting in the biofilm clearance. The bactericide benzalkonium bromide kills bacteria by blocking cell metabolism [27]. 

In this study, the antimicrobial effect on *P. aeruginosa* was enhanced by both copper ions and the bactericide (benzalkonium bromide). The results in Table S1 showed that the presence of bactericide did not enhance the Cu release from Cu-DSS, which indicated that the synergistic effect of the bactericide and 2205-CuDSS was not dependent on the increased amount of Cu released by the bactericide. In previous reports, 2205-Cu DSS was found to be resistant to *P. aeruginosa* in the marine environment. In this study, a small amount of bactericide was combined with 2205-Cu DSS, and the antibacterial activity was significantly improved. This work provides a new idea for enhancing the antibacterial activity of metal materials. From previous studies, it can be found that most antibiotics are used to resist the growth of *P. aeruginosa* [7], and the long-term use of antibiotics will bring a series of bacterial-drug-resistance problems. The combined use of 2205-CuDSS and bactericides can enhance the antibacterial efficiency and reduce the dose of bactericides, while the use of a small amount of bactericides can also alleviate the burden of super drug-resistant bacteria.

## 5. Conclusions

The antibacterial efficacy of 2205-Cu DSS combined with benzalkonium bromide against *P. aeruginosa* was better than that of benzalkonium bromide or 2205-Cu DSS alone. The combination of the antibacterial metal with a chemical bactericide reduced the adhesion of *P. aeruginosa* on the sample surface, which achieved a better germicidal efficacy by inhibiting cell multiplication and killing the bacteria. The combination of the antibacterial metal with a chemical bactericide provides a plausible strategy to mitigate the excessive application of non-eco-friendly bactericides.

## Figures and Tables

**Table 1:** Chemical compositions of 2205 DSS and 2205-Cu DSS.

Component	Si	Mn	P	S	Ni	Cr	Mo	Cu	N	Fe
DSS (wt%)	0.49	1.12	0.03	<0.001	5.82	23.12	3.14	-	0.19	Balance
Cu-DSS (wt%)	0.05	0.02	0.005	0.0032	6.01	23.53	2.85	3.01	0.21	Balance

## Supplementary Materials

The following supporting information can be downloaded at: https://www.mdpi.com/article/10.3390/microorganisms11030711/s1, Figure S1: The amount of ATP of different groups. standard deviations were from three independent experiments. *** *p* < 0. 001 (sample vs. the 2205 DSS group) (n − 3).; Table S1: Cu^2+^ release from 2205-Cu DDS at different exposure time in LB.
