# Supplementary material for: Synergistic Antibacterial Activity of Benzalkonium Bromide and Cu-Bearing Duplex Stainless Steel against Pseudomonas aeruginosa
Source: Microorganisms. 2023 Mar 9;11(3):711. doi: 10.3390/microorganisms11030711 (PMC10057093; doi:10.3390/microorganisms11030711)
Supplement: Supplementary file 1 [file microorganisms-11-00711-s001.zip › microorganisms-2161135-SI.docx]

Supplementary data


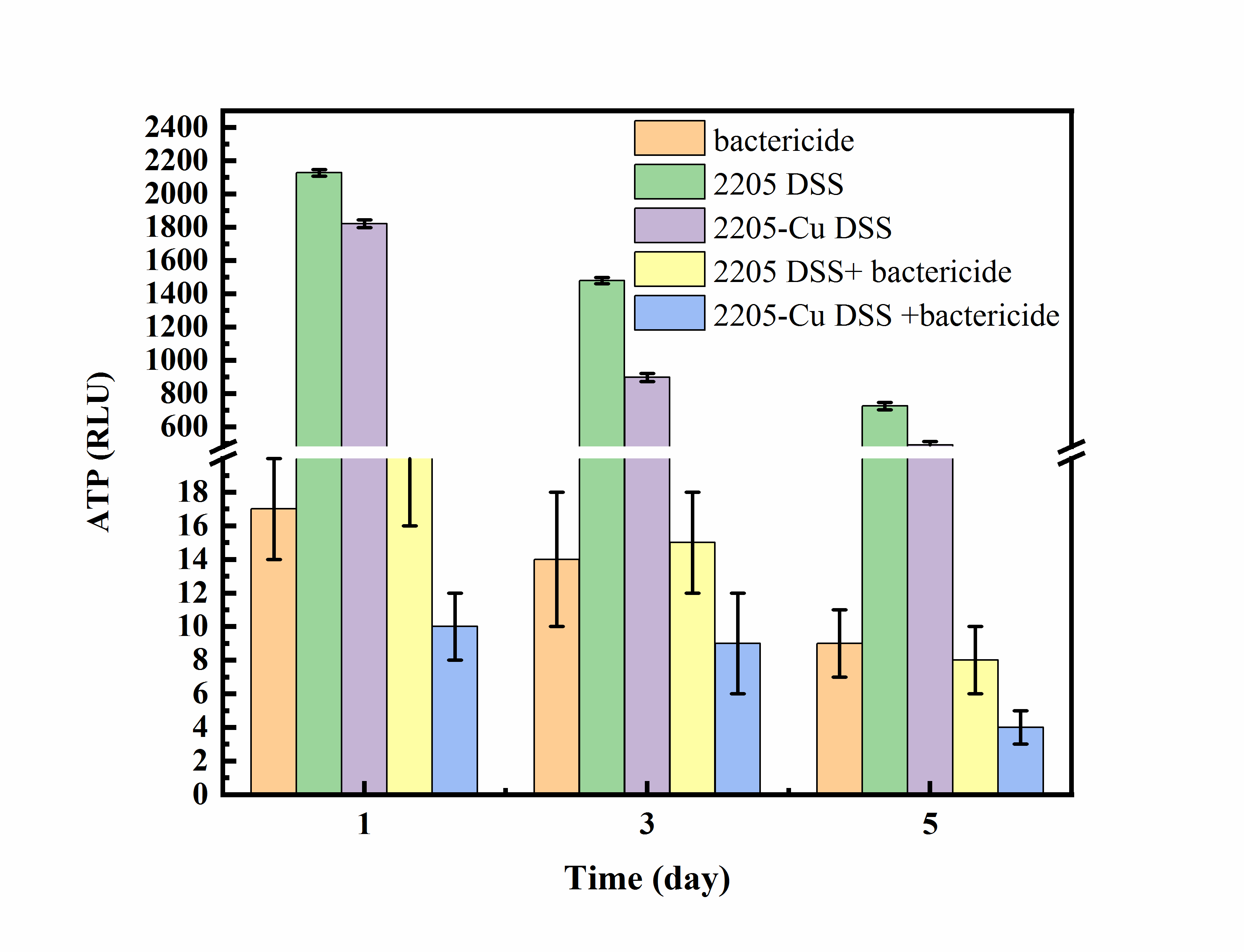


*******

*******

*******

Figure S1. The amount of ATP of different groups. standard deviations were from three independent experiments. * * **P* < 0. 001 (sample *vs* the 2205 DSS group). (n-3).

Table S1

| **Time (days)** | **1** | **3** | **5** |
| --- | --- | --- | --- |
| **With bactericide (** Cu^2+^**+ concentration (ppb))** | **20.9±1.0** | **24.6±1.2** | **32.9±4.7** |
| **Without bactericide (** Cu^2+^ **concentration (ppb))** | **20.1±1.3** | **25.5±1.0** | **33.5±2.9** |

Cu^2+^ release from 2205-Cu DDS at different exposure time in LB.
